# Efficacy and Tolerability of Two Novel “Standard of Care” Treatments—Intranasal Esketamine Versus Intravenous Ketamine—for Treatment-Resistant Depression in Naturalistic Clinical Practice: Protocol for a Pilot Observational Study

**DOI:** 10.2196/34711

**Published:** 2022-05-23

**Authors:** Gilmar Gutierrez, Joshua Rosenblat, Emily Hawken, Jennifer Swainson, Gustavo Vazquez

**Affiliations:** 1 School of Medicine Faculty of Health Sciences Queen's University Kingston, ON Canada; 2 Canadian Rapid Treatment Center of Excellence Toronto, ON Canada; 3 Department of Psychiatry Faculty of Health Sciences Queen's University Kingston, ON Canada; 4 Department of Psychiatry University of Alberta Edmonton, AB Canada

**Keywords:** major depressive disorder, antidepressant, treatment, intervention, pharmacology, pharmacological, Treatment resistant depression, esketamine, ketamine, psychopharmacotherapy, pharmacotherapy, depression, depressive disorder, treatment, observational study

## Abstract

**Background:**

Intravenous (IV) ketamine and intranasal (IN) esketamine have been studied as novel alternatives to manage treatment-resistant depression (TRD). The objective of this observational pilot study is to compare the real-world effectiveness and tolerability of IV ketamine and IN esketamine in the management of unipolar TRD.

**Objective:**

To compare the effectiveness (primary outcome measure) and tolerability (secondary outcome measure) of racemic ketamine and esketamine in the management of TRD in adults and provide an expert qualitative commentary on the application of IV ketamine and IN esketamine in clinical practice (exploratory objective), focusing on the recruitment process, patient retention, effectiveness, and tolerability of the treatments.

**Methods:**

This is a multicenter prospective observational study of naturalistic clinical practice. We expect to recruit 10 patients per treatment arm—IV ketamine or IN esketamine per center (2 centers, total 40 subjects). Patients experiencing moderate to severe TRD and who are candidates for receiving low-dose IV ketamine treatments or IN esketamine as part of their standard-of-care treatments will be recruited. We will measure the effectiveness of each treatment arm by measuring the severity of depression symptoms using the Montgomery and Åsberg Depression Rating Scale; tolerability, side effects, and the appearance of dissociation symptoms using the simplified 6-item version of the Clinician Administered Dissociative Symptom Scale (CADSS-6); and potential for abuse using a Likeability and Craving Questionnaire. Logistic regression will examine odds ratios, number needed to treat for response and remission, number needed to harm, and likelihood to be helped or harmed of each treatment. Covariate analysis will assess the impact of site and demographic variables on treatment efficacy.

**Results:**

This observational trial was approved by the Queen’s University Health Science and Affiliated Teaching Hospital’s Research Ethics Board in February 2021. The two research centers involved have started patient recruitment. Our research center (Providence Care Hospital, Kingston, Ontario) has recruited 9 patients so far. We expect to finalize data gathering by August 2022. The manuscript is expected to be published by December 2022.

**Conclusions:**

We hypothesize that both treatments will have comparable rapid and robust antidepressant effects and similar tolerability profiles in a real-world setting for the management of TRD.

**International Registered Report Identifier (IRRID):**

DERR1-10.2196/34711

## Introduction

Racemic ketamine and its enantiomer esketamine have been studied as novel alternatives for treatment-resistant depression (TRD) in major depressive disorder (MDD) and bipolar depression [1-3]. Studies with intravenous (IV) racemic ketamine have demonstrated rapid and potent reduction of depressive symptoms after the administration of a single subanesthetic dose (response rate=3.01, 95% CI 1.96-4.62; remission rate=3.70, 95% CI 2.28-6.01) [4-8]. Studies with intranasal (IN) esketamine have shown effectiveness in the treatment of TRD for acute and long-term maintenance use (response rate=1.38, 95% CI 1.06-1.79; remission rate=1.47, 95% CI 1.12-1.94) [8,9]. These constitute relevant findings as MDD ranks among the top mental health disorders in the United States and Canada [10-12]. Further, this is the number 1 cause of disability from mental health disorder and number 2 overall, causing substantial losses in quality of life and productivity [11,12]. Unfortunately, most of the published data on the effectiveness and tolerability of these innovative treatments come from clinical trials and not from head-to-head comparative observations of naturalistic clinical practice [8,9,13]. Observational studies are important as they can provide a clearer picture of how these innovative therapies behave in a real-world setting, instead of the more ideal and tightly controlled environment of a clinical trial. Thus, this research study aims to fill this gap, through a head-to-head comparison of IV ketamine to IN esketamine treatment in a Canada-wide multicenter (Providence Care hospital in Kingston, Ontario, and the Canadian Rapid Treatment Center of Excellence in Toronto, Mississauga, and Ottawa) pilot evaluation of naturalistic clinical practice.

Owing to the significant prevalence and overall mental and physical health impact of TRD [14-16], the understanding of the effectiveness and tolerability of IV ketamine and IN esketamine is paramount to the field. Applying meta-analytic comparison across clinical trials, IV ketamine has been shown to be more efficacious and tolerable than IN esketamine for the treatment of depression [8]. Additionally, research has shown that aside from its antidepressant properties, IV ketamine can rapidly reduce suicidal ideation in patients with depression [7,17-20], but IN esketamine has not [21,22]. However, it is important to note that IN esketamine has been approved by the US Food and Drug Administration (FDA) for the treatment of TRD and has clinical trials supporting its use with more long-term data and larger sample sizes than IV ketamine [8]. Moreover, it has been proposed that IN esketamine has a higher affinity for the N-methyl-D-aspartate (NMDA) receptors, is less psychomimetic, and has a greater analgesic and anesthetic effect than the R-ketamine enantiomer. This allows the use of lower doses, potentially with less risk of side effects than IV ketamine [23,24].

Therefore, our primary objective will be to compare the effectiveness of IV ketamine and IN esketamine to each achieve clinical response and remission based on the changes of depression scores according to the Montgomery and Åsberg Depression Rating Scale (MADRS) from baseline to study end point in patients with MDD experiencing TRD. We will use the clinical response to treatment to calculate the number needed to treat (NNT). Our secondary objective will be to evaluate the tolerability of these treatments by systematically assessing the appearance of the most commonly reported adverse effects. We will calculate the number needed to harm (NNH) and the likelihood of being helped or harmed (LHH) [25]. Finally, looking at the recruitment process of patients with MDD experiencing TRD across Canada and the effectiveness and tolerability of these treatments, we will comment on the feasibility of implementing IV ketamine and IN esketamine as standard-of-care treatments as our exploratory outcome objective. The understanding of these novel treatments continues to evolve; thus, our study will supplement the available literature and guide clinical practice in support of either or both treatments in terms of effectiveness and tolerability.

## Methods

### Study Design

**Figure 1:**
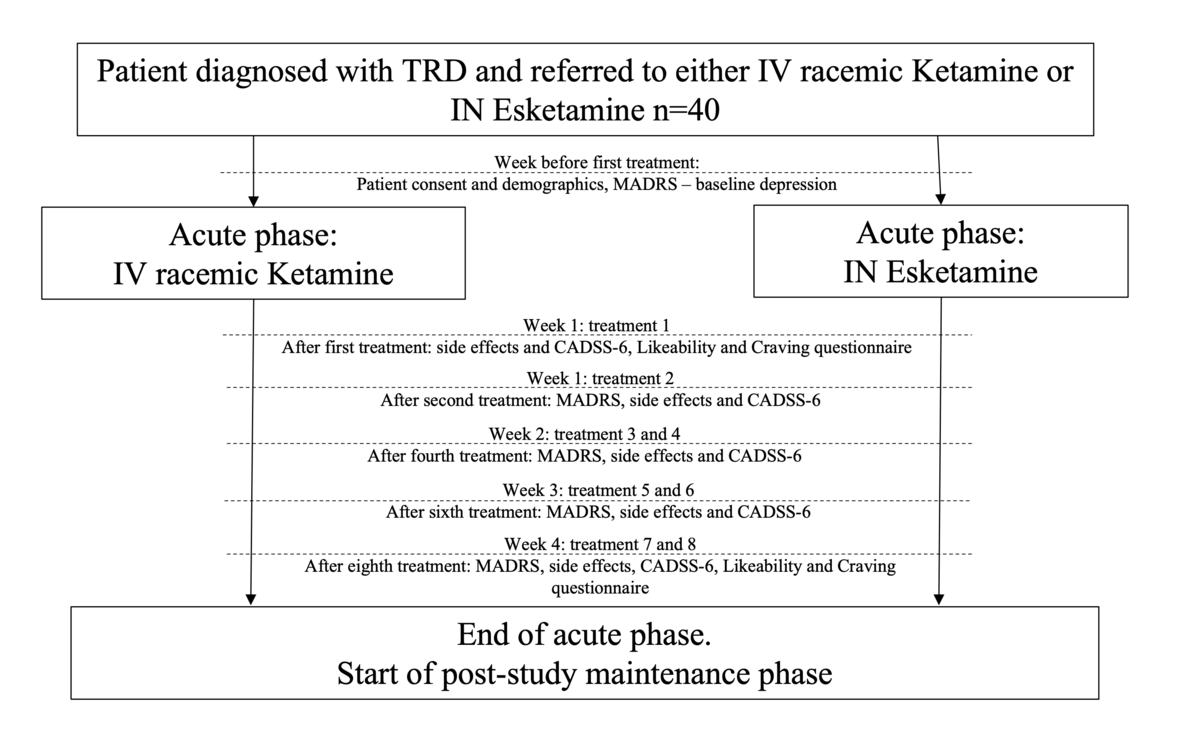
Study design overview. This study involves 4 weeks of acute ketamine treatment. During the acute phase the patients will have up to 2 sessions per week. Baseline MADRS total scores will be measured on the week before the first treatment. Then, MADRS, side effects and CADSS6 will be done up to 24 hours after the second, fourth, sixth and eighth sessions (side effects and CADSS-6 done up to 24 hours after the first session too). Likeability and Craving questionnaire will be done up to 24 hours after the first and eighth, sessions. CADSS: Clinician Administered Dissociative Symptom Scale; IN: intranasal; IV: Intravenous; MADRS: Montgomery and Åsberg Depression Rating Scale; TRD: treatment-resistant depression.

This is a multicenter (Providence Care hospital in Kingston, and the Canadian Rapid Treatment Center of Excellence in Toronto, Mississauga, and Ottawa) observational pilot study of naturalistic clinical practice comparing the efficacy and tolerability of IV ketamine and IN esketamine in the management of patients with MDD and those with TRD [26]. Both IV ketamine and IN esketamine treatments are administered in accordance with the standards-of-care national clinical guidelines [27-29] and pharmaceutical’s monograph approved by Health Canada [30], respectively. Participants with moderate to severe MDD experiencing TRD who have been assessed and approved to receive these innovative therapies will go through an acute phase of treatment of up to 8 sessions (IV ketamine or IN esketamine) for 4 weeks. After the acute phase, patients may continue their treatments per each center’s clinical protocols and will not be followed as part of this research project. Depression symptom severity will be assessed through the treatment course using the MADRS [31]. Side effects will be recorded through treatment sessions as well, using a checklist of common side effects [32], and we will use the Simplified 6-Item Clinician Administered Dissociative Symptoms Scale (CADSS-6) [33] to assess the severity of dissociative symptoms during treatment (a common side effect in NMDA glutamatergic treatments). Further, potential for abuse of these treatments will be evaluated using a Likeability and Craving Questionnaire (LCQ) (Dr Jennifer Swainson and Dr Jay Wang, University of Alberta, Edmonton). Figure 1 presents a summary of the experimental design.

### Participants and Recruitment

Patients (n=40), aged 18-65 years, with MDD as determined by Diagnostic and Statistical Manual of Mental Disorders, 5th edition, diagnostic criteria and characterized as moderate to severe TRD (baseline MADRS score≥20 and experienced at least 2 failed antidepressant trials of adequate dose and duration [26,34]) will be assessed to receive IV ketamine or IN esketamine treatment as part of their standard-of-care treatment. Exclusion criteria are reporting any active substance abuse, symptoms of psychosis, diagnosis of bipolar disorder, or personality disorder as the primary diagnosis (as defined by the patient’s psychiatrist or primary care provider), uncontrolled hypertension, previous negative reaction to racemic ketamine or esketamine, and being currently pregnant or breastfeeding. These patients will be consented to be followed through their treatment course, as part of our research study. This is an observational open-label pilot study, which usually requires a minimum sample of 10-12 participants per treatment arm, considering a main study with a power estimate of 90% and CI of 95% [35-40]. Hence, we consider that our sample size (N=40) is sufficient.

### Demographics

After obtaining consent to be enrolled in this study, a demographics questionnaire collecting the following information will be completed: age, gender, marital status, living situation, education, employment, health plan or means of accessing either treatment, drug use, and diagnosed medical or psychiatric comorbidities. The aforementioned factors can influence disease and treatment progression in this patient population [41,42]. For instance, socioeconomic differences could promote or hinder the improvement of depression symptoms because of this treatment. Demographic data will be used to stratify our results to better understand their impact on treatment efficacy.

### Rapid-Acting Glutamatergic Treatments

In the clinic, eligible patients will be offered IN esketamine as part of their standard-of-care treatment for TRD initially as an option. If their health insurance coverage cannot cover the costs of IN esketamine, then patients will be offered IV ketamine. We will observe patients undergoing either IV ketamine or IN esketamine treatments and record their vital signs, any potential side effects during and after administration, and changes in severity of depressive symptoms. For the acute phase, both treatments occur up to twice a week over a period of 4 weeks for a total of 6-8 sessions depending on each of the clinical site’s protocols. Patients may choose to continue their treatments beyond this point in the maintenance phase, but they will not be followed up as part of this study. Patients are asked to have no solid foods or liquids (nil per os) 2 hours prior to treatment. Treatment sessions will occur at each of the 2 centers as part of their ongoing clinical services.

For IV ketamine, an indwelling catheter placed in the nondominant arm is used. This treatment is administered at a subanesthetic dose: 0.5 mg/kg up to 1 mg/kg (depending on the level of response of the patient to the treatment) over 40 minutes. Administration is carried out with the patient in bed rest, and then the patient is asked to remain in bed for 30 minutes after administration before starting activity as tolerated. Nasal cannula oxygen may be administered if needed using side-stream capnometry monitoring. Pulse, blood pressure, pulse oximetry, and electrocardiography are assessed before the start and through the treatment with IV ketamine. Physiological monitoring data are recorded on a standard anesthesia record beginning 5 minutes before treatment. These patients are discharged after a minimum of 30 minutes post administration, provided that the vital signs have returned to baseline and that the patient is calm, alert, and oriented [27].

IN esketamine is administered as a spray that delivers a 14-mg dose per spray per nostril (28 mg total per device), which the patients self-administer under the supervision of a health care provider. Depending on the response of the patient to the treatment and their clinician’s decision, they may self-administer up to 84 mg of IN esketamine, by using up to 3 devices, waiting 5 minutes between each 28-mg dose, per the esketamine product monograph [30]. Patients are allowed to start activity as tolerated after administration. Nasal cannula oxygen may be administered if needed using side-stream capnometry monitoring. Pulse, blood pressure, and pulse oximetry are assessed before the start and for 30 minutes after the IN spray of esketamine. Physiological monitoring data are recorded on a standard anesthesia record beginning 5 minutes before treatment. Then, these patients are discharged after a minimum of 2 hours post administration, provided that the vital signs have returned to baseline and that the patient is calm, alert, and oriented [27].

For IV ketamine, patients will start therapy at 0.5 mg/kg [28,29,43]. If the patient has a partial response (defined as a decrease in the MADRS total score of between 25% and <50% of the baseline) after the second week of treatment, the dose will be increased to 0.75 mg/kg for IV ketamine. For IN esketamine, the recommended dose titration by the manufacturer and approved by Health Canada will be followed (i.e., 56 mg on the first treatment and from then on 84 mg per treatment) [44,45]. Further dose modifications can be carried out on an individual level as needed, and an average dose throughout the treatment will be reported to account for these changes.

### Outcome Evaluation

#### Overview

Effectiveness and tolerability of the ketamine treatments will be determined through assessment scales (MADRS, side effects checklist, CADSS-6, and LCQ) by a trained interviewer, over the phone, through videoconference, or in person when possible. We will inform the patient about the potential risks associated with a phone or videoconference assessment and obtain consent to continue before starting each assessment. Treatment effectiveness will be assessed using the MADRS [31], measuring baseline severity of depressive symptoms the week before the first session of either treatment (IV ketamine or IN esketamine), and then progression of depression symptoms through the treatment course once per week, up to 24 hours after the second, fourth, sixth, and eighth (study end point) sessions. Tolerability will be assessed by tracking the side effects and potential for abuse of either treatment. We used the side effects checklist (Multimedia Appendix 1) [32] and the CADSS-6 for symptoms of dissociation (a common side effect of ketamine treatment) [33] up to 24 hours after the first, second, fourth, sixth, and eighth sessions. Potential for abuse for either treatment will be determined using the LCQ (Multimedia Appendix 2) up to 24 hours after the first and eighth sessions. Figure 1 provides an overview of the study design and the application of each of the mentioned scales through the treatment course.

#### Primary Outcome Measure

The effectiveness of racemic ketamine and IN esketamine in improving the depression scores will be determined from baseline to the study end point (completion of the treatment course) using the MADRS in patients with an episode of MDD and TRD. Response to treatment will be defined as a minimum reduction of 50% in their baseline depression score. We will calculate the number NNT for response and remission [25] based on this result. Remission from depression is defined as a depression rating of less than or equal to 10 on the MADRS [46]. Improvement in suicidality will be defined as any change in suicidal ideation severity from baseline to study end point using MADRS item 10.

#### Secondary Outcome Measures

We will determine the tolerability of racemic ketamine and esketamine by calculating the NNH and the LHH [25] based on the number of patients who completed the treatment versus dropouts. Completion of treatment will be defined as the proportion of participants who remained in the study until the completion of the treatment course (6-8 sessions depending on each of the clinical site’s protocols). Dropouts will be defined as patients who discontinued the study prematurely owing to any cause and owing to adverse events. Adverse events will be tracked using a checklist of side effects (Multimedia Appendix 1) [32] and dissociation will be assessed using the CADSS-6 [33]. All subjects will complete the LCQ at baseline and the end point to investigate the potential development of craving for these treatments as an adverse event (Multimedia Appendix 2).

#### Exploratory Outcome Measures

We will gather expert commentary from the attending physicians implementing IV ketamine and IN esketamine treatment at their respective collaborating research centers. We will focus on the recruitment process and overall patient experience (each researcher is asked to keep a systematic record of their recruitment process and patient retention). Then, with this information and the assessment of the effectiveness and tolerability of these treatments, we will make a qualitative commentary on the feasibility of implementing IV ketamine and IN esketamine as standard-of-care treatments in naturalistic clinical practice.

### Ethics and Privacy

All components of this study were approved by the Queen’s University Health Sciences and Affiliated Teaching Hospitals Research Ethics Board. To protect their privacy, participants will be given an anonymous and unique code that was used to identify their data through all the assessment measures and data processing and for all purposes of knowledge dissemination (including but not limited to peer-reviewed publications, scientific presentations, grant proposals, and reports). Assessment data will be stored in secured and password-protected laptops for 5 years after the study completion date, and hard copies of consent forms and participants identifying data will be stored on site in secured lockers and destroyed 5 years after study completion. The research team will safeguard the privacy of the participants to the extent permitted by the applicable laws and duty to report. Grounds for breaching confidentiality will include immediate physical risk to the self or others, elder abuse, and child abuse and neglect.

### Data Analysis

The collected data will be analyzed by applying descriptive statistics: mean, median, SD, maximum, and minimum scores for primary and secondary outcome measures and patients’ demographics data. To assess treatment efficacy, changes in depression symptoms as measured with the MADRS with either IV ketamine or IN esketamine treatment and will be tracked and compared over time (1 month of the acute phase) using within-subjects repeated measures ANOVA for each treatment arm. Further, we will calculate the response to treatment as the proportion of patients who reached a minimum reduction of 50% from their baseline depression score by study end point and the remission from depression as the proportion of patients who reported a MADRS total score equal or less than 10 at the study end point [46]. Logistic regression will be used to calculate odds ratios. Moreover, we will calculate the NNT, the NNH, and the LHH: NNT by considering the response to treatment and NNH by considering the proportion of patients who completed the study versus the dropouts and the adverse effects (side effects checklist and dissociation using the CADSS-6) experienced by the patients. LHH will be calculated as the ratio of NNH and NNT [25]. Linear regression will examine the contribution of treatment arm (IV ketamine versus IN esketamine), site, and patients’ demographic data to account for the influence of these variables to our calculation of effectiveness. Adverse events will be reported in terms of frequency through the treatment course. The data will be compared using a Mann-Whitney *U* test to determine if there is a significant difference in the response to the 2 treatments (mean depressive scores, mean dissociation scores, and mean LCQ scores). The effect size of each treatment will be calculated using Cohen *d* [47].

## Results

Since this observational trial was approved by the Queen’s University Health Science and Affiliated Teaching Hospitals Research Ethics Board in February 2021, we began offering patients the opportunity to participate in this study in March 2021 at the Mood Disorders Services in Providence Care Hospital, Kingston. Thus far, we have recruited 9 patients, and we expect to finalize data gathering from the 2 centers by August 2022 and analyze the findings by October 2022 at which point, we will begin our process of knowledge dissemination (including but not limited to peer-reviewed publications, scientific presentations, grant proposals, and reports). The manuscript is expected to be published by December 2022.

## Discussion

### Expected Findings

Both IV ketamine and IN esketamine have been shown separately to be effective and potent at managing symptoms of TRD [1-8]. Meta-analytic indirect comparison of these 2 treatments favored IV ketamine, showing that overall, it has a higher response, higher remission rates, and lower dropouts than IN esketamine. Though it is important to mention that phase 3 studies tend to have smaller effect sizes than phase 2 studies [48], and unlike with ketamine, there are phase 3 studies with esketamine, which may impact this analysis [8]. However, IN esketamine has been recently approved for the management of TRD by the FDA and Health Canada [8] and has several features that can make it a more desirable treatment. For instance, its higher affinity for the NMDA receptor means that it can be administered at lower doses than IV ketamine, and IN administration is more convenient and comfortable for patients than IV administration. However, the high cost of IN esketamine treatment may be a significant limiting factor for patients [20,23,49]. Nevertheless, currently, there are no direct comparisons of these 2 treatments in clinical practice, and most of the information that we have about either treatment comes from clinical trials that usually present an overly idealistic version of real-world clinical practice [8,9,13]. Thus, this research study aims to fill this gap through a head-to-head comparison of racemic low-dose IV ketamine with IN esketamine treatment in a multicenter (Providence Care hospital in Kingston and the Canadian Rapid Treatment Center of Excellence in Toronto, Mississauga, and Ottawa) pilot evaluation of naturalistic clinical practice. Hence, through this study, we aim to guide clinical practice in support of either or both treatments in terms of effectiveness and tolerability.

The two main strengths of this study are that it observes the effectiveness and tolerability of these novel treatments in naturalistic clinical practice, and that both treatments are administered in the same 4 clinics and under similar conditions. Naturalistic clinical administration of IV ketamine and IN esketamine would allow us to better understand how these innovative therapies behave in a real-world setting and with the challenges of administering these treatments in clinical practice, such as scheduling conflicts, managing side effects, and modifying dosing schedules in accordance with symptom improvement and side effects, among others. Further, by having both treatments in the same clinical setting, with minimal changes other than the administration route, we hope to minimize external factors that could confound the effect of the treatments.

Our primary outcome focuses on determining the effectiveness of IV ketamine and IN esketamine on symptoms of depression in patients with MDD experiencing TRD. By applying the MADRS [31], we will track the change in the severity of depressive symptoms and compare the effectiveness of these 2 treatments in terms of clinical response and remission from depression and reduction in suicidal ideation. Clinical response results will be taken into account to calculate the NNT [25] for IV ketamine and IN esketamine. Our secondary outcome focuses on the tolerability of IV ketamine and IN esketamine on patients with MDD experiencing TRD. By applying different measurements focusing on side effects (side effects checklist [32]), including dissociation (CADSS-6 [33]) and potential for abuse or craving (LCQ), we will observe the patients through their treatment course to determine the NNH and the LHH [25]. Our exploratory outcomes will focus on the expert commentary of the attending physicians at each of the collaborating research centers with regard to their recruitment process and patient experience through the treatment course. This information and our assessment of the effectiveness and tolerability of these treatments will be used to make a qualitative commentary on the feasibility of implementing IV ketamine and IN esketamine as standard-of-care treatments in naturalistic clinical practice. From this pilot study, we expect that IN esketamine may be less accessible (owing to cost) and thus less feasible for the average person with TRD. We also expect that both treatments will be similar in terms of effectiveness and tolerability; thus, these results will help inform clinical practice in the use of these novel treatments for the management of TRD.

### Limitations and Future Research

The main limitations in this study are associated with the challenges of real-world clinical practice. We foresee that scheduling conflicts with the patients’ treatment sessions may cause alterations in the acute regimen, and some patients may choose to stop the treatment abruptly. Further, the significant cost and still scarce insurance coverage for IN esketamine treatment means that only some patients will be able to opt to receive this treatment. As a result, we may have biases in the demographics of patients who are able to receive either treatment—this will be analyzed at the end of the study. Another limitation is that owing to COVID-19 restrictions, we have resorted to carrying out the assessment scales over the phone. This represents an important limitation, as a key part of understanding the patients’ mental state is through their body language and their appearance. Furthermore, we are not able to carry out the assessment promptly after the treatment sessions and usually have to wait till the patient gets to their home. This represents a problem, especially for side effect–tracking (including dissociation), as the patient is asked to remember their state several hours after the treatment.

In terms of future direction, as this is a pilot study, we are planning to expand this study to include a comparison with other standard-of-care treatments for MDD and TRD. Further, we are planning to carry out a longer-term follow-up assessment of these patients—for 6 months and 1 year—to further assess the extent of the benefit of these innovative treatments. We will also expand the follow-up assessment to both patients who have continued with routine maintenance and those who have slowly tapered their sessions and may no longer be taking the medication. Through this future study, we could help determine effective ways to apply these treatments in clinical practice to achieve long-lasting results and better understand the potential benefits of these treatments over the long term.

### Conclusions

IV ketamine and IN esketamine are novel, rapid-acting, promising treatments for TRD; hence, we aim to understand whether these treatments are comparable in terms of effectiveness and tolerability in naturalistic clinical practice. We hypothesize that owing to the chemical similarity of these two compounds, they would have comparable effects; thus, we hope to inform clinical practice in support of either or both treatments.

## Appendix

Multimedia Appendix 1 Side Effects Checklist.

Multimedia Appendix 2 Likeability & Craving Questionnaire.

## Abbreviations

CADSS-6: Simplified 6-Item Clinician Administered Dissociative Symptoms Scale
FDA: US Food and Drug Administration
IN: intranasal
IV: intravenous
LCQ: Likeability and Craving Questionnaire
LHH: likelihood to be helped or harmed
MADRS: Montgomery and Åsberg Depression Rating Scale
MDD: major depressive disorder
NMDA: N-methyl-D-aspartate
NNH: number needed to harm
NNT: number needed to treat
RR: response rate
TRD: treatment resistant depression
