# Supplementary material for: Efficacy and Tolerability of Two Novel “Standard of Care” Treatments—Intranasal Esketamine Versus Intravenous Ketamine—for Treatment-Resistant Depression in Naturalistic Clinical Practice: Protocol for a Pilot Observational Study
Source: JMIR Res Protoc. 2022 May 23;11(5):e34711. doi: 10.2196/34711 (PMC9171596; doi:10.2196/34711)
Supplement: Multimedia Appendix 1 [file resprot_v11i5e34711_app1.docx]

**MULTIMEDIA APPENDIX 1. Side Effects Checklist.**

| **Side Effects Checklist** | YES | NO |
| --- | --- | --- |
| Elevated Heart Rate |  |  |
| Feelings of Anxiety |  |  |
| Faintness/Light Headedness |  |  |
| Sedation/Drowsiness |  |  |
| Nausea |  |  |
| Blurred Vision |  |  |
| Increased Blood Pressure |  |  |
| Dizziness |  |  |
| Headache |  |  |
