# Supplementary material for: Efficacy and Tolerability of Two Novel “Standard of Care” Treatments—Intranasal Esketamine Versus Intravenous Ketamine—for Treatment-Resistant Depression in Naturalistic Clinical Practice: Protocol for a Pilot Observational Study
Source: JMIR Res Protoc. 2022 May 23;11(5):e34711. doi: 10.2196/34711 (PMC9171596; doi:10.2196/34711)

**Multimedia appendix 2**

**Likeability & Craving Questionnaire**

**Instruction**

In this questionnaire, you will be asked to provide responses according to the degree of your experience. To do so, you will be asked to put a vertical line on a scale from 0 – 10, which is meant to represent the degree to which you agree or disagree with the question. In addition, you will be asked to put your answer in the box to the right of the scale. An example is provided below. Please read the scale carefully to understand what the numbers on the scales represent.

*Sample Response:*

*Overall, my liking for vanilla ice cream is: (0 = strong dislike, 5 = neutral, 10 = strong liking)*


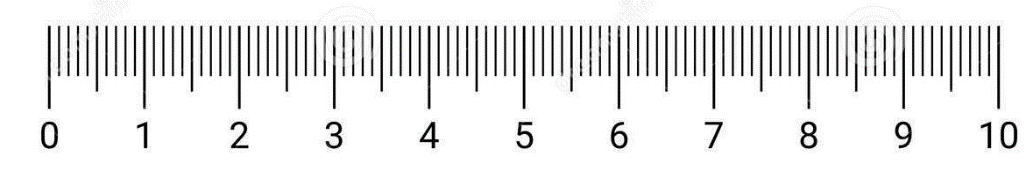


8.7

1. **Likeability for ketamine**

“Liking” can be understood as the degree one looks forward to the positive psychoactive effects of a substance. Examples of these effects can include experiences such as feeling “high”, feeling detached from one’s body, feelings of floating, feelings of wellbeing, experience of strong emotions, or increased sensitivity to the senses such as light or sound. **These can be an effect of ketamine treatment immediately after it is given, and “liking” refers to these effects, NOT any antidepressant or anti-suicidal effects.**

Please assess your degree of likeability for ***ketamine’s dissociative effects*** (**NOT ANTIDEPRESSANT EFFECTS)** per the following questions by putting one vertical line on each of the following scale.

1. Overall, my liking for ketamine is:

(0 = strong dislike; 5 = neutral; 10 = strong liking)


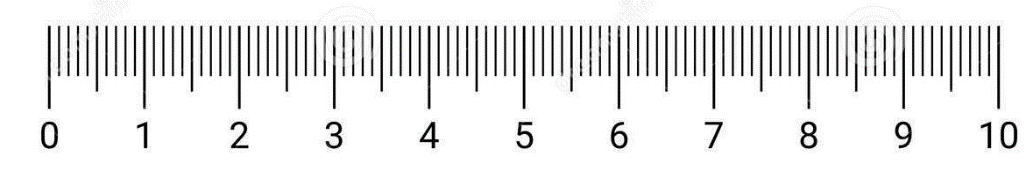


1. **Craving for ketamine**

“Craving” for a substance can be understood as an intense desire and motivation to acquire and to use a drug. Please assess your degree of craving for ketamine per the following questions by putting one vertical line on each of the following scale.

1. Overall, how much do you crave ketamine treatments for effects **other than antidepressant effects?**

(0 = absolutely no craving, 5 = neutral, 10 = constantly craving)


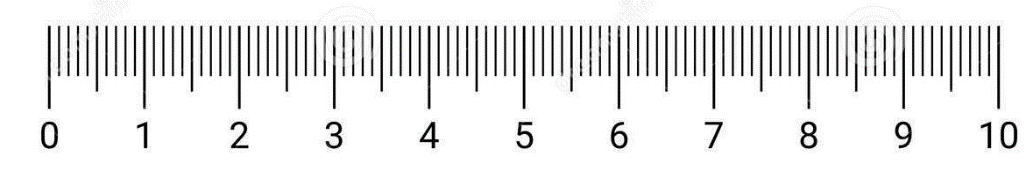


1. How much do you desire to use ketamine i**n greater amounts or higher frequently than your psychiatrist is prescribing for reasons other than hoping for more antidepressant effect**? This does not need to mean you have done so, but that you experience desire in between treatments to experience the psychoactive effects of ketamine.

(0 = absolutely no craving, 5 = neutral, 10 = very strong craving)


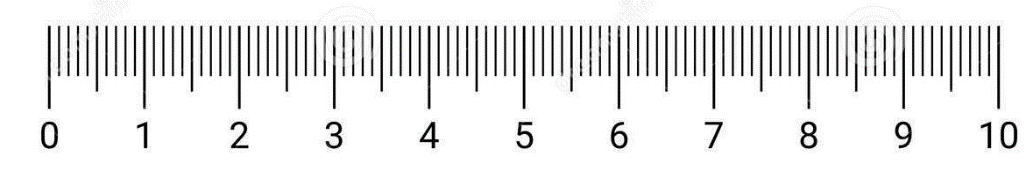

Supplement: Multimedia Appendix 2 [file resprot_v11i5e34711_app2.docx]
